# Detection, Characterization and Evolution of Internal Repeats in Chitinases of Known 3-D Structure

**DOI:** 10.1371/journal.pone.0091915

**Published:** 2014-03-17

**Authors:** Manigandan Sivaji, Vinoth Sadasivam, Jayabalan Narayanasamy, Selvaraj Samuel, Chuanzhu Fan

**Affiliations:** 1 Department of Plant Science, Bharathidasan University, Tiruchirappalli, India; 2 Department of Biological Sciences, Wayne State University, Detroit, Michigan, United States of America; 3 Department of Bioinformatics, Bharathidasan University, Tiruchirappalli, India; Indian Institute of Science, India

## Abstract

Chitinase proteins have evolved and diversified almost in all organisms ranging from prokaryotes to eukaryotes. During evolution, internal repeats may appear in amino acid sequences of proteins which alter the structural and functional features. Here we deciphered the internal repeats from Chitinase and characterized the structural similarities between them. Out of 24 diverse Chitinase sequences selected, six sequences (2CJL, 2DSK, 2XVP, 2Z37, 3EBV and 3HBE) did not contain any internal repeats of amino acid sequences. Ten sequences contained repeats of length <50, and the remaining 8 sequences contained repeat length between 50 and 100 residues. Two Chitinase sequences, 1ITX and 3SIM, were found to be structurally similar when analyzed using secondary structure of Chitinase from secondary and 3-Dimensional structure database of Protein Data Bank. Internal repeats of 3N17 and 1O6I were also involved in the ligand-binding site of those Chitinase proteins, respectively. Our analyses enhance our understanding towards the identification of structural characteristics of internal repeats in Chitinase proteins.

## Introduction

Chitin is one of the most abundant biopolymer in nature and is made up of an insoluble homopolymer of β-1,4 linked *N*-acetyl glucosamine (GlcNAc) units [1]. Chitin serves a morphological structural role in arthropods, including crustaceans and insects, as well as mollusks, nematodes, and worms. It is also found in fungi, making up from less than 1% to more than 40% of the cell wall, depending on the species [2]. Chitinases are hydrolytic enzymes that break down the glycosidic bonds in chitin. Chitinases are occurring in organisms that need to either reshape their own chitin or dissolve and digest the chitin of other invading fungi and animals.

Chitin has not been found in mammals. Nevertheless, several mammalian proteins with homology to fungal, bacterial, or plant Chitinase have been identified [3]. All Chitinases have been recognized to play important roles in self-defense against pathogens [4]. Most recently, however, some Chitinases have been found to appear in response to environmental stresses, such as cold, drought, and high salt concentration [4]. Other Chitinases are reported to participate in important physiological processes of plants, such as embryogenesis and ethylene synthesis [4]. The variable effectiveness of specific Chitinases against different pathogens and the existence of microbial Chitinase inhibitors led to the hypothesis that Chitinases may co-evolve with fungi in response to variation in pathogen defenses against chitinolytic activity [5].

The majority of protein sequences is aperiodic and usually has globular 3D structures carrying a number of various functions. The foremost efforts of researchers were devoted to these types of proteins and as a result, significant progress has been made in the development of bioinformatics tools for their analysis [6], [7]. However, proteins also contain a large portion of periodic sequences representing arrays of repeats that are directly adjacent to each other [8].

Intragenic duplications of genetic material have important biological roles because of their protein sequence and structural consequences [9]. Bioinformatics tools are important for analysis of protein repeats with emphasis on the sequences, 3D structures, and sequence–structure relationship as well as highlighting successful strategies for the prediction of the protein structure [10]. These tandem repeats are considerably diverse, ranging from the repetition of a single amino acid to domains of 100 or more residues. They are ubiquitous in genomes and occur in at least 14% of all proteins [11]. Before analysis of repeats, it just needs to score protein sequences in multiple sequence alignment. Common methods (e.g. the dot matrix method) for detection of similarity depend on pairwise alignment of sequences [12]. The abundance of natural structured proteins with tandem repeats is inversely correlated with the repeat perfection. The chance to find natural structured proteins in Protein Data Bank (PDB) (http://www.rcsb.org/pdb) increases with a decrease in the level of repeat perfection [10].

When a certain threshold of the conserved residues in the repeat is exceeded, the repetitive regions of proteins are predominantly disordered and the main reason of residue conservation in tandem repeats may due to the change from a structural to an evolutionary one [13]. Hence, internal repeats in Chitinase involved in diversification of Chitinases with different structural and functional properties and it may also play role in quick evolution of Chitinase in all organisms. Repetitive sequences apparently formed after the prokaryotic-eukaryotic divergence by a mechanism with weak length-dependence such as recombination. Repetitive proteins evolve quicker than non-repetitive proteins [11]. Protein repeats have highlighted the multi-functionality of repeat types, their structural differences, and their proliferations in different evolutionary lineages. One likely reason for their evolutionary success is that repeat-containing proteins are relatively “cheap” to evolve. By this we mean that large and thermodynamically stable proteins may be arisen by the simple expedient of intragenic duplications, rather than the more complex processes of *de novo* α-helix and β-sheet creation [14].

## Materials and Methods

### Selected sequences of Chitinase

Chitinase sequences were obtained from PDB [15]. ]. Among 147 Chitinase sequences of known structure retrieved from PDB, 34 sequences were selected based on 50% sequence identity, which includes both eukaryotic and prokaryotic Chitinase sequences. Among the obtained 34 sequences, ten did not have the Chitinase domain and these were excluded from further analysis. The remaining 24 Chitinases sequences were subsequently used to analyze for detection of internal repeats and secondary structure (Table 1).

**Table 1:** List of amino acid sequences of Chitinase protein used in the present study.

PDB ID	Species	Division	Length of protein (amino acids)
3FND	*Bacteroides thetaiotaomicron*	Bacteria	312
3IAN	*Lactococcus lactis*	Bacteria	321
3N17	*Bacillus cereus*	Bacteria	333
3QOK	*Klebsiella pneumonia*	Bacteria	420
3ARX	*Vibrio harveyi*	Bacteria	584
2CJL	*Streptomyces coelicolor*	Bacteria	204
1WVV	*Streptomyces griseus*	Bacteria	265
1ITX	*Bacillus circulans*	Bacteria	419
1KFW	*Arthrobacter sp.*	Bacteria	435
1O6I	*Serratia marcescens*	Bacteria	499
3EBV	*Streptomyces coelicolor*	Bacteria	302
3OA5	*Yersinia entomophaga*	Bacteria	543
3G6M	*Clonostachys rosea*	Fungi	406
2Y8V	*Aspergillus fumigatus*	Fungi	290
2XVP	*Aspergillus fumigatus*	Fungi	310
3HBE	*Picea abies*	Plant	204
3ALF	*Nicotiana tobaccum*	Plant	353
2Z37	*Brassica juncea*	Plant	244
2DKV	*Oryza sativa* L. *japonica*	Plant	309
3CQL	*Carica papaya*	Plant	243
3SIM	*Crocus vernus*	Plant	275
2DSK	*Pyrococcus furiosus*	Archaea	311
3BXW	*Homo sapiens*	Animal	393
1WB0	*Homo sapiens*	Animal	445

### Detection of internal repeats using RADAR

We used RADAR (Rapid Automatic Detection and Alignment of Repeats) (http://www.ebi.ac.uk/Tools/pfa/radar/) to identify internal repeats in protein sequences. Many large proteins evolved from internal duplication and many internal sequence repeats correspond to functional and structural units. RADAR uses an automatic algorithm by segmenting query sequence into repeats and identifies short composition biased as well as gapped approximate repeats. Complex repeat architectures involve many different types of repeats in query sequence [16]. The segmentation procedure has three steps: (i) repeat length is determined by the spacing between suboptimal self-alignment traces; (ii) repeat borders are optimized to yield a maximal integer number of repeats, and (iii) distant repeats are validated by iterative profile alignment.

### Computing the % identity between the repeat sequences detected by RADAR

As RADAR gives only a Z-score between the repeats, we computed the % identity between each repeat pair or the tandem repeats (more than a pair of repeats) in a protein using the Smith-Waterman server available at the European Bioinformatics Institute (http://www.ebi.ac.uk/Tools/psa/emboss_water/) [17], [18].

### Evaluation 3-D structural similarity of the Chitinases

The structural relatedness of the proteins involves consideration of average root-mean-square deviation (RMSD) of Cα atoms and Z-score between structures. The structural similarity of the 24 Chitinase structures was carried using PDBeFOLD server [19]. The PDB structures were downloaded from RCSB website (http://www.rcsb.org/pdb) and the PDB coordinates were uploaded to the server for finding structural similarity. PDBeFold structural similarity searches were conducted using WWW interface at http://www.ebi.ac.uk/msd-srv/ssm/.

### Visualization using RasMol

RasMol is a molecular graphics visualization tool which is used for primary depiction and exploration of biological macromolecular structures, such as those found in the PDB [20]. The secondary structure region which is corresponding to internal repeat sequences was used for structural analysis. The secondary structure of the Chitinase was retrieved from PDB and then the repeated region was detected as structure. The repeated region was visualized in 3-D structure using RasMol software and the repeated sequences were separated and visualized using RasMol. PDB file of all Chitinase sequences downloaded from PDB were edited and extracted the repeated amino acid sequence in separate files for comparison in RasMol. PDB files can be downloaded for visualization in RasMol.

### Multiple sequence alignment and phylogenetic tree

Multiple sequence alignment was carried out using ClustalW [21] and MUSCLE [22]. The phylogenetic tree was constructed using Neighbor Joining method implemented in MEGA [23]. The bootstrap analysis with 10,000 replicates was used to assess the robustness of the branches.

## Results and Discussion

### Internal repeats analysis

Of 24 selected sequences of Chitinase from various organisms, RADAR was performed to detect the internal repeats. Six out of 24 sequences (2CJL, 2DSK, 2XVP, 2Z37, 3EBV and 3HBE) do not contain any internal repeats. The repeats in the remaining sequences vary from 2 repeats per amino acid sequence of Chitinase proteins. Some Chitinases with more than two repeats were also observed. For example, 3IAN, 3N17, 3ARX, 2DKV, 1ITX, and 1WB0 contain two repeated regions; 3ARX and 3ALF contain two tandem repeats and 3QOK contains four tandem repeats. Length of amino acid residues of Chitinase proteins which are identified in repeat region also varies. Ten sequences contained repeats of length <50, and the remaining 8 sequences contained repeat length between 50 and 100 residues. Table 2 shows the % identity obtained between pairs of repeats or tandem repeats in a given Chitinase. Analysis of the extent of sequence identity between the internal repeats reveal that in general shorter repeats have higher % identity while longer repeats have low % identity. This reveals that the repeats have diverged considerably after the duplication event.

**Table 2:** List of internal repeats identified in different Chitinase sequences available in the Protein Data Bank with % identity between the repeats and RMSD.

PDB ID	Organism name	# of repeats	# of segments	Repeat region	% identity	Length	RMSD (Å)
3FND	*Bacteroides thetaiotaomicron*	1	2	68–81/200–215	50.0	14	1.80
3G6M	*Clonostachys rosea*	1	2	39–120/145–238	31.3	93	3.07
3IAN	*Lactococcus lactis*	2	2	46–76/155–180	28.6	26	2.04
			2	24–37/87–100	42.9	14	-
3N17	*Bacillus cereus*	2	2	133–192/233–291	32.7	55	1.24
			2	119–129/204–214	54.5	11	0.70
				10–83/86–125	33.3		-
				10–83/293–357	30.0		2.6
3QOK	*Klebsiella pneumonia*	1	4	10–83/358–395	27.8	74	-
				86–125/293–357	50.0		-
				86–125/358–395	35.0		-
				293–357/358–395	22.5		2.81
				166–222/234–263	42.9		-
3ALF	*Nicotiana tobaccum*	1	3	166–222/274–331	31.7	57	1.95
				234–263/274–331	40.0		2.4
			2	291–338/384–437	30.4	47	3.16
3ARX	*Vibrio harveyi*	2		70–115/465–532	25.0		-
			3	70–115/536–574	27.1	46	-
				465–532/536–574	24.2		1.55
3BXW	*Homo sapiens*	1	2	62–136/209–280	38.5	67	3.26
2Y8V	*Aspergillus fumigatus*	1	2	161–192/244–280	24.3	32	0.96
2DKV	*Oryza sativa L. japonica*	2	2	31–54/140–163	45.5	23	-
			2	121–133/224–238	46.2	13	1.59
1WVV	*Streptomyces griseus*	1	2	4–73/189–255	19.0	66	-
1ITX	*Bacillus circulans*	2	2	33–114/235–317	27.0	81	3.40
			2	159–213/360–428	31.5	54	3.8
		2	2	72–119/120–236	32.7	69	1.4
			2	72–119/238–292	27.1		-
1KFW	*Arthrobacter sp.*			120–236/238–292	20.7		3.8
		3	3	340–349/436–443	62.5	9	-
				359–386/388–412	43.5	23	-
1O6I	*Serratia marcescens*	1	2	201–215/413–428	50.0	15	-
1WB0	*Homo sapiens*	2	2	98–113/185–195	43.8	11	0.58
			2	247–270/352–374	33.3	23	2.76
3CQL	*Carica papaya*	1	2	15–45/87–113	30.8	25	-
			2	48–69/410–438	31.0	22	-
3OA5	*Yersinia entomophaga*	4	2	75–91/128–146	80.0	17	-
			2	104–113/202–211	50.0	10	-
			2	219–233/462–475	40.0	14	-
3SIM	*Crocus vernus*	1	2	156–178/187–212	32.0	23	1.36

### Fold distribution of Chitinases

The Chitinases appear to be very diverse in terms of sequence and yet adopt only a limited number of folds. Analysis of the folds of the Chitinases using CATH database (http://www. cathdb.info) reveals that they belong to two major folds, namely, i) Triosephosphate isomerase (TIM) barrel fold and ii) Endochitinase fold. TIM barrel is a conserved protein fold consisting of eight α-helices and eight parallel β–strands that alternate along the peptide backbone [24]. Among the 24 Chitinases considered, 18 of them belong to the TIM barrel fold and 6 belong to the Endochitinase fold.

### Inter-repeat % sequence identity among TIM barrel fold sequences

As a number of TIM barrel fold Chitinases contain long repeats, we assessed the % sequence identity across the various repeats in this fold using the Emboss Waterman – Smith local alignment algorithm. Quite interestingly, the Chitinases including 1ITX, 3ARX, and 1KFW all shared >40% sequence identity in the repeat regions (Table 3). Analysis of the presence of DXDXE functional motif in Chitinase sequences reveals that this motif was conserved in all sequences of the TIM barrel fold. The rest of the sequences which belong to the Endochitinase fold did not contain the above motif. Interestingly, this motif was also present in the RADAR detected internal repeat region of 1ITX, 3ARX, 3G6M and 1KFW. The inter-sequence repeat analysis carried out between the Chitinases containing internal repeats and those without internal repeats showed scores less than 25% identity.

**Table 3:** Inter – repeat % identity across different TIM fold Chitinase sequences.

PDB Code	Internal repeat segments	Description	3ARX	1ITX	1KFW
	1	% of identity	23.4	25.4	29
3G6M	39–120	Aligned Sequence	139–248	17–127	2–122
	2	% of identity	29.4	37.7	27.2
	145–238	Aligned Sequence	269–368	142–245	137–259
	1	% of identity		41.0	55.6
	291–338	Aligned Sequence		149–186	175–201
	2	% of identity		45.8	52.6
	384–237	Aligned Sequence		240–263	262–280
3ARX	1	% of identity		30.0	37.5
	70–115	Aligned Sequence		325–344	335–350
	2	% of identity		33.3	30.2
	465–532	Aligned Sequence		326–353	243–285
	3	% of identity		43.2	27
	536–574	Aligned Sequence		367–403	369–405
	1	% of identity	30.6		31.8
	33–114	Aligned Sequence	118–178		1–69
	2	% of identity	34.4		36.4
1ITX	235–317	Aligned Sequence	326–386		235–317/216–284
	1	% of identity	46.2		40.8
	159–213	Aligned Sequence	159–213/250–300		159–213/123–193
	2	% of identity	31.9		35.0
	360–428	Aligned Sequence	474–545		360–428/341–399
	1	% of identity	36.8	45.5	
1KFW	72–119	Aligned Sequence	238–337	72–119/116–214	
	2	% of identity	33.3	40.0	
	238–292	Aligned Sequence	352–387	238–292/239–267	

### 3-D structural similarity between the Chitinases

The RMSD and Z-scores obtained for pair-wise structural alignments obtained between the Chitinases belonging to the TIM fold and Endochitinase fold are given in Table S1 and Table S2 respectively. In general all the structures retain similar three-dimensional structures as revealed by the low RMSD values and high Z-scores. Among the Chitinases belonging to the TIM fold, the structures of 3G6M, 1O6I, 3ARX, 3OA5, 1ITX, 1KFW, 1WBO, 3QOK, and 3ALF shared an RMSD <2.0 angstrom (Å). Quite interestingly, proteins belonging to this set with 3G6M, 3ARX, 1ITX, and 1KFW share reasonable inter-repeat % identity between them (Table 3). Other proteins belonging to the TIM fold share RMSD >2.0 Å (Table S1).

Among the Chitinases belonging to the Endochitinase fold, most of them share RMSD <2.0 Å whereas the pairs 3HBE *vs* 2CJL, 3HBE *vs* 1WVV show low RMSD. It is interesting to point out that among these three proteins, both 2CJL and 3HBE do not have any repeats and 3-D structural similarity within repeats (intra-repeat) in Chitinases (Table 2).

**Figure 1:**
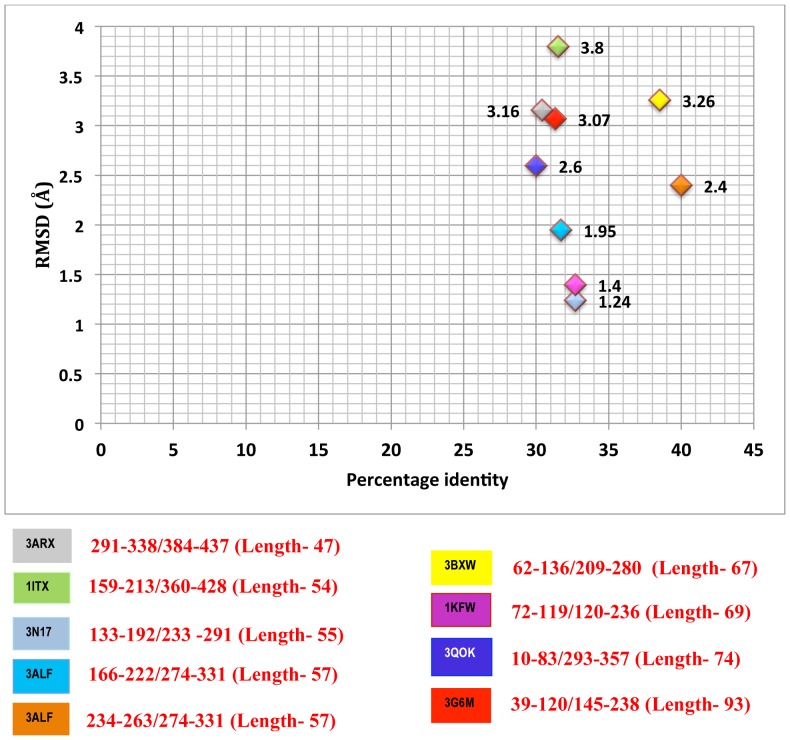
Relationship between RMSD values and percentage identity of TIM fold intra-repeats.

Surprisingly, in many cases the repeats are too divergent to be identified as similar structure based on visual analysis. Structural alignment of these repeats may uncover more similar members and provide an objective way to identify truly dissimilar structural repeats. Hence structural superposition of repeats of Chitinases belonging to the TIM barrel fold was carried out. The results reveal that the RMSD between superposed repeats ranges from 0.70 Å to 3.8 Å (Table 2). Ignoring repeats of short length, the variation in RMSD with % sequence identity of intra-repeats in 8 Chitinases belonging to the TIM barrel fold is plotted in Figure 1. The results demonstrate that repeats in 3ARX, 1ITX, 3BXW and 3G6M show larger deviation in structure as shown by RMSD >2.5 Å. Repeats in 3N17, 3ALF, 1KFW and 3QOK show lower structural divergence (RMSD<2.5 Å).

### Structural visualization of internal repeats in Chitinase

**Figure 2:**
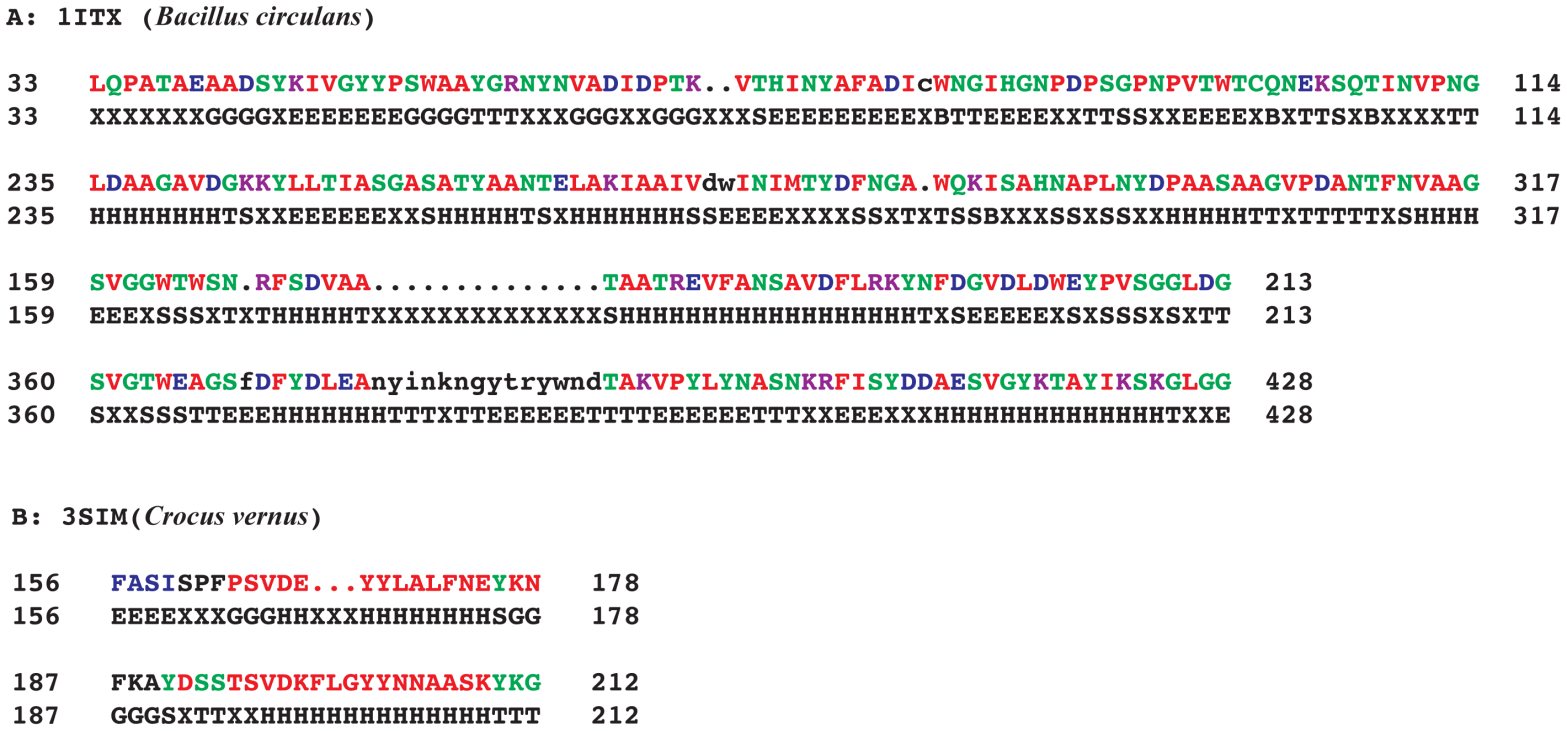
Internal repeats with their corresponding secondary structure. The internal repeats identified using RADAR was used to compare the internal repeats with its secondary structure using secondary structure database of PDB. The structure revealed the secondary structure as follows: T: Turn, E: Beta strand, G: 3/10 helix, B: Beta bridge, S: Bend, H: Alpha-Helix. These five repeats showed similar secondary structures between the internal repeats of corresponding Chitinase sequences. A: 1ITX (*Bacillus circulans*) shows the repeat regions 33-114, 235-317 and 159-213, 360 - 428 and their corresponding DSSP secondary structure assigned from PDB; B: 3SIM (*Crocus vernus*) shows internal repeat regions from 156-178 and 187-212 and its corresponding secondary structure assignments.

**Figure 3:**
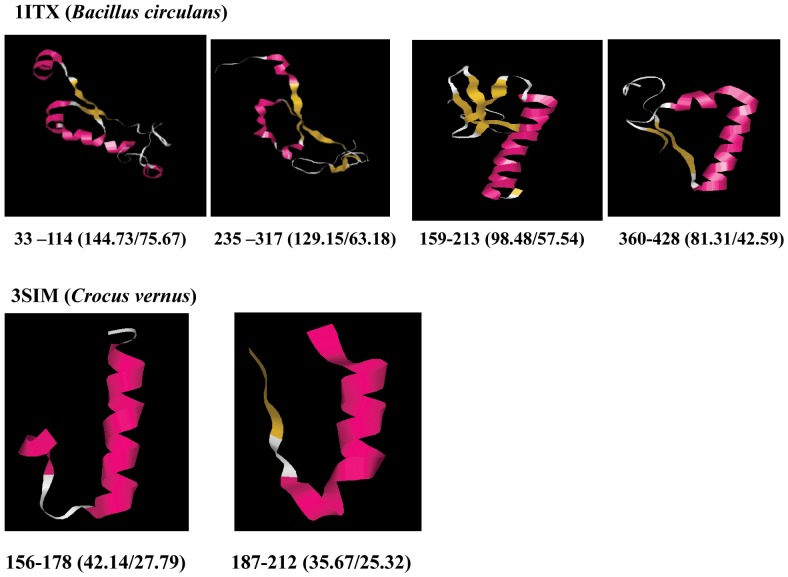
Visualization of internal repeats in 3-D view using RasMol.

The internal repeats identified using RADAR were used to separate the secondary structure of those repeat regions from whole secondary structure of that particular Chitinase protein sequence. When comparing the identified internal repeat amino acid sequence to corresponding secondary structure, the visual secondary structures in repeated region of Chitinase sequences are resolved. On the basis of structural similarity of secondary structural elements in the repeat regions, similarity in the 3-D structure was further analyzed. The structural arrangement in the repeated region between two repeats is easy for structural comparison. The 1ITX (2 β strands and 1 turn) and 3SIM (1 turn and 1 α helix) showed similar secondary and tertiary structural arrangements (Figures 2 and 3). In other cases, although repeats could be identified based on sequence similarity, no structural similarity could be observed.

### Analysis of amino acid residues of repeat segments present in ligand binding site

**Figure 4:**
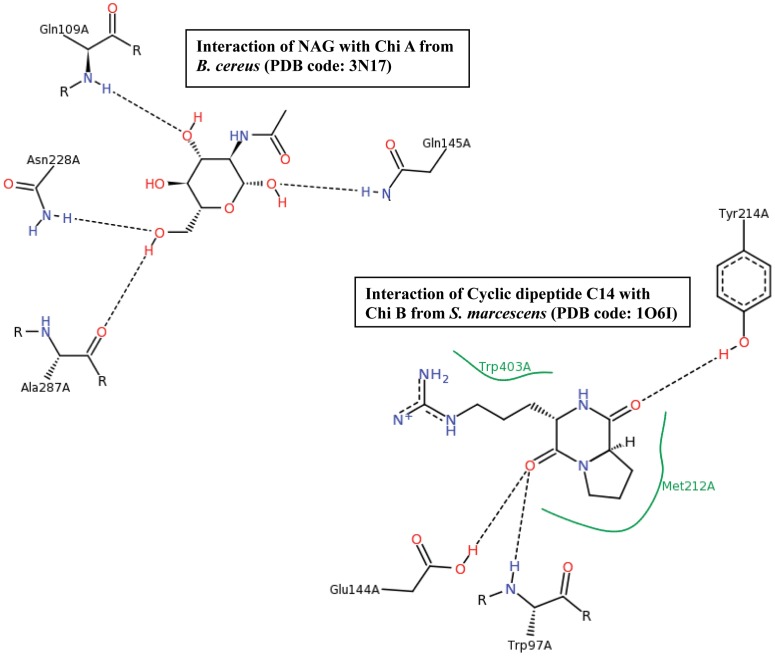
Ligand-protein interaction in 3N17 (NAG - Chi A) and 1O6I (Cyclic Dipeptide C14 - Chi B).

We further analyzed the involvement of residues in the repeat segments in the binding of ligands. Excluding the binding of very small ligands such as sulphate, phosphate and glycerol, we observed binding of N-acetyl-d-glucosamine (NAG) in 3N17 and that of a cyclic dipeptide C14 in 1O6I. In 3N17 Chi A, apart from residues Gln 109 and Ala 287, Gln 145 from repeat 1 and Asn 228 from repeat 2 are involved in binding of NAG. Like-wise, residues Met 212 and Tyr 214 in 1O6I from repeat 1 are involved in the binding of cyclic dipeptide C14. The other binding site residues namely, Trp 97, Glu 144 and Trp 403 are not part of the repeated segment (Figure 4).

### Alignment scores

Alignment scores of all selected Chitinase sequences generated for the multiple sequence alignment are shown in Table S3. Among the 24 sequences, those from *Bacteroides thetaiotaomicron* (3FND), *Homo sapiens* (3BXW), *Aspergillus fumigates* (2XVP), (2Y8V), *Crocus vernus* (3SIM), showed alignment scores ≤20 (Table S3).

### Multiple sequence alignment and phylogenetic analysis of Chitinases

**Figure 5:**
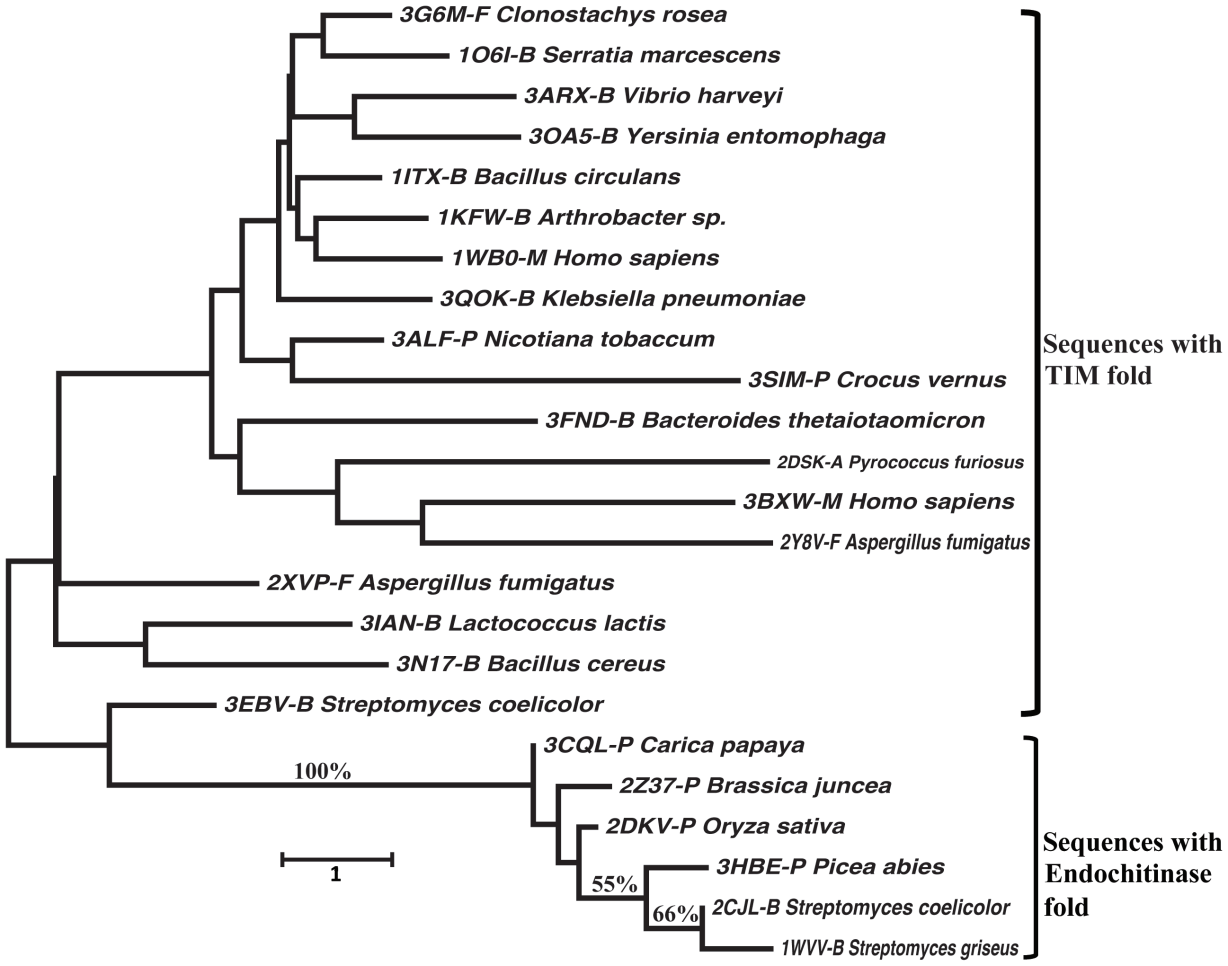
Phylogenetic analysis of selected 24 Chitinases for fold analysis. Bootstrap support value (%) >50 is showed above branch.

The multiple sequence alignment for 18 TIM barrel fold Chitinases and 6 Endochitinase fold Chitinases considered in the study are showed in Figure S1 and Figure S2, respectively. Wherever present, the repeat segments are marked in the sequences. As the Chitinases considered belong to a diverse set of sequences, no uniformity in the location of repeats could be observed. The phylogenetic tree revealed two major clusters with 100% bootstrap support, one having all Chitinases belonging to the TIM barrel fold and another having the Endochitinase fold (Figure 5). We also performed phylogenetic analysis for each fold Chitinases. The phylogenetic relationships of Chinitases with Endochitinase fold are similar to the combined phylogenetic analysis (Figure S3), but relationships of Chinitases with TIM barrel fold show some discrepancy to the combined analysis (Figure S4), which suggested the sequence divergence is higher for TIM barrel Chinitases.

## Conclusions

The sequence comparison between different organism of both eukaryotes and prokaryotes reveals occurrence of internal repeats in Chitinase protein in most cases. The Chitinases considered here adopt two major folds, namely, the TIM barrel fold and the Endochitinase fold. There are huge differences in the number of internal repeats and number of amino acid residues present in each internal repeat. The present study reveals that in general intra-protein repeats of length >50 show low % identity, reflecting the considerable divergence that has taken place after the duplication event. Repeats in some Chitinase belonging to the TIM barrel fold also show considerable structural divergence as revealed by higher RMSD values. Also the sequence location of the repeats is not uniform. Quite interestingly, in spite of divergence at the sequence level, almost of all the structures considered in the present study retain similar three-dimensional folding as revealed by the low RMSD values. Many large proteins have evolved by internal duplication and many internal sequence repeats correspond to functional and structural units [16]. The present study suggests that the internal repeats present in Chitinases do not disturb their stability or alter their structures or function.

## Supporting Information

Figure S1 Multiple sequence alignment of 18 TIM barrel fold Chitinases with the repeats regions marked.
(PDF)

Figure S2 Multiple sequence alignment of 6 Endochitinase fold Chitinases with the repeats regions marked.
(PDF)

Figure S3 Phylogenetic relationship of Endochitinase fold Chitinases. Bootstrap support value (%) >50 is showed above branch.
(TIF)

Figure S4 Phylogenetic relationship of TIM barrel fold Chitinases. Bootstrap support value (%) >50 is showed above branch.
(TIF)

Table S1 Alignment scores of different pairs of Chitinases.
(PDF)

Table S2 RMSD and Z-scores of structural superposition of proteins belonging to the TIM fold.
(PDF)

Table S3 RMSD and Z-scores of structural superposition of proteins belonging to the Endochitinase fold.
(PDF)
