# Supplementary material for: Detection, Characterization and Evolution of Internal Repeats in Chitinases of Known 3-D Structure
Source: PLoS One. 2014 Mar 17;9(3):e91915. doi: 10.1371/journal.pone.0091915 (PMC3956812; doi:10.1371/journal.pone.0091915)
Supplement: Figure S1 — Multiple sequence alignment of 18 TIM barrel fold Chitinases with the repeats regions marked. (PDF) [file pone.0091915.s001.pdf]

Figure S1. Multiple sequence alignment of 18 TIM barrel fold Chitinases with the repeats regions marked with different color

```
                                        10        20        30        40        50        60        70        80        90       100
                                ....|....|....|....|....|....|....|....|....|....|....|....|....|....|....|....|....|....|....|....|
3G6M:F|C.rosea Nematophagous f  ----------------------------------------------------------------------------------------------------
1O6I:B|S.Marcescens CHITINASE   ----------------------------------------------------------------------------------------------------
3ARX:B|V.harveyi Chitinase A    APTAPSIDMYGSNNLQFSKIELAMETTSGYNDMVKYHELAKIKVKFNQWSGTSGDTYNVYFDGVKVATGAITGSQTTASFEYGQGGLYQMEIEACDATGC
3OA5:B|Y.entomophaga Chi1 Chit  --------------------------------------MSYYHHHHHHLESTSLYKKAGFENLYFQGSGAMEKEEKSNLIYDKDPGYVWDN NECEGAAE
1ITX:B|B.circulans Chitinase A  ----------------------------------------------------------------------------------------------------
1KFW:B|Arthrobacter sp. Psychr  ----------------------------------------------------------------------------------------------------
1WB0:M|H.sapiens HUMAN CHITINA  ----------------------------------------------------------------------------------------------------
3QOK:B|K.pneumoniae Putative c  ----------------------------------------------------------------------------------------------------
3ALF:P|N.tobaccum Class V Chit  ----------------------------------------------------------------------------------------------------
3SIM:P|C.vernus Family 18 Chit  ----------------------------------------------------------------------------------------------------
3FND:B|B.thetaiotaomicron       ----------------------------------------------------------------------------------------------------
2DSK:A|P.furiosus Hyperthermop  ----------------------------------------------------------------------------------------------------
3BXW:M|H.sapiens Chitinase-Lik  ----------------------------------------------------------------------------------------------------
2Y8V:F|A.fumigatus PDBID|CHAIN  ----------------------------------------------------------------------------------------------------
2XVP:F|A.fumigatus CHIA1        ----------------------------------------------------------------------------------------------------
3IAN:B|L.lactis Chitinase   su  ----------------------------------------------------------------------------------------------------
3N17:B|B.cereus Chitinase  NCT  ----------------------------------------------------------------------------------------------------
3EBV:B|S.coelicolor Putative C  ----------------------------------------------------------------------------------------------------

                                       110       120       130       140       150       160       170       180       190       200
                                ....|....|....|....|....|....|....|....|....|....|....|....|....|....|....|....|....|....|....|....|
3G6M:F|C.rosea Nematophagous f  -----------------RATPRMEDLASTDLSTRATGSINAVYFTNWGIYGRN---FQPADLQAS----K---------ILHVLYSFMNLR---------
1O6I:B|S.Marcescens CHITINASE   ---------------------------------MSTRKAVIGYYFIPTNQINN---YTETDTS-------------------------------------
3ARX:B|V.harveyi Chitinase A    SKSAPVEITIADTDGSHLKPLTMNVDPNNKSYNTDPSIVMGTYFVEWGIYGRD---YTVDNMPVDN-------------LTHILYGFIPICG------PN
3OA5:B|Y.entomophaga Chi1 Chit  ETYQELNYEPSISAD LTWTPT LA TVFNTYEDDDDFNVLCYFTDWSQYDPRIINKEIRDTGGRSADIL LNTPDG PF  LIYSFGGLIGDKKYSADG
1ITX:B|B.circulans Chitinase A  --------------------------LQPATAEAADSYKIVGYYPSWAAYGRN---YNVADIDPT----K---------VTHINYAFADICWNGIHGNPD
1KFW:B|Arthrobacter sp. Psychr  -----------------------------PLTSTVNGYRNVGYFAQWGVYGRA---FQAKQLDVSGTAKN----------LTHINYSFGNINNQTLTCFMA
1WB0:M|H.sapiens HUMAN CHITINA  -------------------------------------AKLVCYFTNWAQYRQGEARFLPKDLDPS-----------------------------------
3QOK:B|K.pneumoniae Putative c  ------------SNAMKRLPLLAALPLLCASALSAQPLMSVGYFNGGG VTAG----PGG I KL-----------------------------------
3ALF:P|N.tobaccum Class V Chit  -----------------------------------MQNVKGGYWFKDSGLALN-------NIDST-----------------------------------
3SIM:P|C.vernus Family 18 Chit  ----------------------------------TLFVEYIGYPLFSGVKFSDVP--INPHITK------------------------------------
3FND:B|B.thetaiotaomicron       ----------------------------------MSLKVVIGYLALDDWEFES----LFPTIEWK-----------------------------------
2DSK:A|P.furiosus Hyperthermop  --------------------------------GPNANPIPEHFFAPYIDMSLSVHKPLVEYAKLTG----------------------------------
3BXW:M|H.sapiens Chitinase-Lik  ----------------------MRTLFNLLWLALACSPVHTTLSKSDAKKAASKTLLEKSQFSDK-----------------------------------
2Y8V:F|A.fumigatus PDBID|CHAIN  -------------------------GPMPPVPGRPEHRRVICYHQTLCPNRGDYVSVLPLVKN-------------------------------------
2XVP:F|A.fumigatus CHIA1        ------------------------------------FSNLAIYWGQGPNQLRLSHFCQETSLDIIN----------------------------------
3IAN:B|L.lactis Chitinase   su  ---------------------------------MSLDKVLVGYWHNWKSTGKD---GYKGGSSADFN---------------------------------
3N17:B|B.cereus Chitinase  NCT  -------------------------------ANNLGSKLLVGYWHNFDNGTGI---IKLKDVS-------------------------------------
3EBV:B|S.coelicolor Putative C  --------------------------------MSL-KHAVTGYWQNFNNGATV---QKISDVP-------------------------------------

                                       210       220       230       240       250       260       270       280       290       300
                                ....|....|....|....|....|....|....|....|....|....|....|....|....|....|....|....|....|....|....|....|
3G6M:F|C.rosea Nematophagous f  -------VDGTVYSGDTYADLE------KHYSDDS----------------WNDIGTNAYGCVKQLYKLKKANRSLKIMLSIGGWTWSTN-------FPA
1O6I:B|S.Marcescens CHITINASE   -VVPFPVSNITPAKAKQLTHIN---FSFLDINSNLECAW-----------DPATNDAKARDVVNRLTALKAHNPSLRIMFSIGGWYYSNDLGVSHANYVN
3ARX:B|V.harveyi Chitinase A    ESVKSVGGNSFNALQTACRGVNDYEVVIHDPWAAYQKSFP---------QAGHEYSTPIKGNYAMLMALKQRNPDLKIIPSIGGWTLSDP--------FY
3OA5:B|Y.entomophaga Chi1 Chit  NASIAVRLGVATDPDDAIANHKGKTIPVDPDGAVLASINCGFT WEAGDANERYNQE A GLLGGF LLHEADKELEFSLSIGGWSMSGLFS-------E
1ITX:B|B.circulans Chitinase A  PSGPNPVTWTCQNEKSQTINVPNGTIVLGDPWIDTGKTF-----------AGDTWDQPIAGNINQLNKLKQTNPNLKTIISVGGWTWSN-------RFSD
1KFW:B|Arthrobacter sp. Psychr  NKAQGTGPNGSDGAGDAWADFG------MGYAADKSVSG-----------KADTWDQPLAGSFNQLKQLKAKNPKLKVMISLGGWTWSKN-------FSK
1WB0:M|H.sapiens HUMAN CHITINA  ---------LCTHLIYAFAGMT-------NHQLSTTEWN-------------------DETLYQEFNGLKKMNPKLKTLLAIGGWNFGTQK------FTD
3QOK:B|K.pneumoniae Putative c  -------VRQITHLNYSFGLIYN -- K  TNAALK PAH---------LH IWLSPKVQA LQKLPALRKQNP LKVLLSVGGWGARGFS--------G
3ALF:P|N.tobaccum Class V Chit  ---------LFTHLFCAFADLN--------PQLNQLIIS----------------PENQDSFRQFTSTVQRKNPSVKTFLSIAGGRANST------AYGI
3SIM:P|C.vernus Family 18 Chit  -------FQFVLSFAVDYTASS--------PHTSTNGKFN-------------VFWDSSILGPDQISAIKSSHPNVRVAVSLGGASVGSNT-----VQFQ
3FND:B|B.thetaiotaomicron       ---------YLTHINASFARVK--------ADGTLNINP--------------------VRKRIESVRETAHKHNVKILISLAKNSPGEFT--------T
2DSK:A|P.furiosus Hyperthermop  ----------TKYFTLAFILYS-------SVYNGPAWAG------------------SIPLEKFVDEVRELREIGGEVIIAFGGAVGPYLC---------
3BXW:M|H.sapiens Chitinase-Lik  ---------PVQDRGLVVTDLK-------AESVVLEHRS-------------------YCSAKARDRHFAGDVLGYVTPWNSHGYDVTKVFG------SK
2Y8V:F|A.fumigatus PDBID|CHAIN  -------NTGVTHIIIAAFHLN-------EDPGHITLNDD--------------PPDHEMYNPLWAEVPVLKRSGVKVMGMLGGAAQGSYR--------C
2XVP:F|A.fumigatus CHIA1        -------IGFINYFPDMSPGHWPGSNFGNQCDGSVYVTNDG-----------VVTKLLSGCHQIMEDIPICQAAGKKVLLSIGGAYPPDQS-------IL
3IAN:B|L.lactis Chitinase   su  ---LSSTQEGYNVINVSFMKTP-------EGQTLPTFKP----------------YNKTDTEFRAEISKLNAE-GKSVLIALGGADAHIE----------
3N17:B|B.cereus Chitinase  NCT  --------PKWDVINVSFGETG-------GDRSTVEF--------------SPVYG--TDADFKSDISYLKSK-GKKVVLSIGGQNGVVL----------
3EBV:B|S.coelicolor Putative C  --------SAYDIIAVAFADAT-------TTPGAVTFNL-----------DSAGLGGYTVDQFKADVRAKQAA-GKKVIISVGGEKGTVS----------

                                       310       320       330       340       350       360       370       380       390       400
                                ....|....|....|....|....|....|....|....|....|....|....|....|....|....|....|....|....|....|....|....|
3G6M:F|C.rosea Nematophagous f  AASTEATRATFAKTAVEFMKDWG----------------FDGIDVDWEYPASET-----------DANNMVLLLQRVRQELDSYSATYAN---GYHFQLS
1O6I:B|S.Marcescens CHITINASE   AVKTPASRAKFAQSCVRIMKDYG----------------FDGVDIDWEYPQAAEVDG------------FIAALQEIRTLLNQQTITDGRQALPYQLTIA
```

```
3ARX:B|V.harveyi Chitinase A    DFVDKKNRDTFVASVKKFLKTWK---------------FYDGVDIDWEFPGGGGAAADKG-DPVNDGPAYIALMRELRVMLDELEAETGR-----TYELT
3OA5:B|Y.entomophaga Chi1 Chit  IAKDEILRTNFVEGIKDFFQRFP---------------MFSHLDIDWEYPGSIGAGNPNS-------PDDGANFAILIQQITDAKISNLKG-----ISIA
1ITX:B|B.circulans Chitinase A  VAATAATREVFANSAVDFLRKYN---------------FDGVDLDWEYPVSGGLDGNS--KRPEDKQNYTLLLSKIREKLDAAGAVDGK---KYLLTIA
1KFW:B|Arthrobacter sp. Psychr  AAATEASRQKLVSSCIDLYIKGNLPNFEGRGGAGAAAGIFDGIDIDWEWPGTNSGLAGNGVDTVNDRANFKALLAEFRKQLDAYGSTNNK---KYVLSAF
1WB0:M|H.sapiens HUMAN CHITINA  MVATANNRQTFVNSAIRFLRKYS---------------FDGLDLDWEYPGSQGSPAVD-------KERFTTLVQDLANAFQQEAQTSGKE--RLLLSAA
3QOK:B|K.pneumoniae Putative c  AAATAESRAVFIRSAQKIIQQYG---------------LDGIDLDWEFPVNGAWGLVAS---------QPADRDNFTALLKSLREAVGE----QKLVTI
3ALF:P|N.tobaccum Class V Chit  MARQPNSRKSFIDSSIRLARQLG---------------FHGLDLDWEYPLSAADMTN-----------LGTLLNEWRTAINTEARNSGR---AALLTA
3SIM:P|C.vernus Family 18 Chit  AASVDSWVSNAVTSLTRIIQRYN---------------LDGIDIDYEHFQN-----------------TDKNTFAECIGRLITTLKK-----NGVIS
3FND:B|B.thetaiotaomicron       AINDPKARKELIQQIIAFTKEYK---------------LDGFDIDYEEYDN-----------------WDKNFPSLLVFARGLYLAKEK-----NMLMT
2DSK:A|P.furiosus Hyperthermop  --QQASTPEQLAEWYIKVIDTYN---------------ATYLDFDIEAGIDAD-----------------KLADALLIVQRERPWVKFS----FTLPSD
3BXW:M|H.sapiens Chitinase-Lik  FTQISPVWLQLKRRGREMFEVTG---------------LHDVDQGWMRAVRKHAKG----------LHIVPRLLFEDWTYDDFRNVLDSEDEIEELSKT
2Y8V:F|A.fumigatus PDBID|CHAIN  LDGDQEKFERYYQPLLAMVRRHQ---------------LDGLDLDVEEEMS--------------------LPGIIRLIDRLKLDLGD-----DFIIT
2XVP:F|A.fumigatus CHIA1        SEDSAVAFATFLWGAFGPVAEGWEGPRPFG------DVVVDGFDFDIEHNGGFG---------------YATMVNTFRQYFNQVPE---------RKFYL
3IAN:B|L.lactis Chitinase   su  --LKKSQESDFVNEIIRLVDTYG---------------FDGLDIDLEQAAIEAADN------------QTVIPSALKKVKDHYRKDGK-----NFMIT
3N17:B|B.cereus Chitinase  NCT  -LPDNAAKDRFINSIQSLIDKYG---------------FDGIDIDLQSGIYLNGNDTN------FKNPTTPQIVNLISAIRTISDHYGP-----DFLLS
3EBV:B|S.coelicolor Putative C  -VNSSASATNFANSVYSVMREYG---------------FDGVDIDLENGL----------------NPT-----YMTQALRALSAKAGP-----DMILT

                                        410       420       430       440       450       460       470       480       490       500
                                ....|....|....|....|....|....|....|....|....|....|....|....|....|....|....|....|....|....|....|....|
3G6M:F|C.rosea Nematophagous f  IAAPAGPSHYNVLKLAQLGSVLDNINLMAYDYAG-SWD-SVSGHQTNLYPSTSNP----------------------SSTPFSTKAAVDAYIAAG-VPAS
1O6I:B|S.Marcescens CHITINASE   GAGGAFFLSRYYSKLAQIVAPLDYINLMTYDLAG-PWEKVTNHQAALFGDAAGPTFYNALREANLGWSWEELTRAFPSPFSLTVDAAVQQHLMMEGVPSA
3ARX:B|V.harveyi Chitinase A    SAIGVGYDKIEDVDYADAVQYMDYIFAMTYDFYG-GWNNVPGHQTALYCGSFMRPGQCDGGGVDE-------NGEPYKGPAYTADNGIQLLLAQG-VPAN
3OA5:B|Y.entomophaga Chi1 Chit  SSADPAKIDAANIP-ALMDAGVTGINLMTYDFFTLGDGKLSHHTNIYRDPSDVYS-----------------------KYSIDDAVTHLIDEKKVDPK
1ITX:B|B.circulans Chitinase A  SGASATYAAN--TELAKIAAIVDWINIMTYDFNG-AWQKISAHNAPLNYDPAASAAG------------------VPDANTFNVAAGAQGHLDAG-VPAA
1KFW:B|Arthrobacter sp. Psychr  LPANPADIDAGGWDDPANFKSLDFGSIQGYDLHG-AWNPTLTGHQANLYDDPADPRA--------------------PSKKFSADKAVKKYLAAG-IDPK
1WB0:M|H.sapiens HUMAN CHITINA  VPAGQTYVDAG-YEVDKIAQNLDFVNLMAYDFHG-SWEKVTGHNSPLYKRQEESG----------------------AAASLNVDAAVQQWLQKG-TPAS
3QOK:B|K.pneumoniae Putative c  AVGANAESPKSWVDVKAVAPVLNYINLMTYDMAYGTQYFNSNLYDSSHWPTVAAAD----------------------KYSADFVVNNYLAAG-LKPS
3ALF:P|N.tobaccum Class V Chit  AVSNSPRVNGLNYPVESLARNIDWINLMAYDFYGPNWSPSQTNSHAQLFDPVN-------------------HVSGSDGINAWIQAG-VPTK
3SIM:P|C.vernus Family 18 Chit  FASISPFPSVDEYYLALFNEYKNAINHINYQFKA--------------------------------------YDSSTSVDKFLGYYNNAAS
3FND:B|B.thetaiotaomicron       CAVNSRWLNYG----TEWEQYFDYINLMSYDRGAFTDKPVQHAS-------------------------------YDDFVKDLKYWNEQCRASKS
2DSK:A|P.furiosus Hyperthermop  PGIGLAGGYGIIETMAKKGVRVDRVNPMTMDYYWTPSNAENAIKVAEN----------------------------VFRQLKQIYPEKSDEEI
3BXW:M|H.sapiens Chitinase-Lik  VVQVAKNQHFDGFVVEVWNQLLSQKRVGLIHMLTHLAEALHQARLLALLVIPP------------------AITPGTDQLGMFTHKEFEQLA
2Y8V:F|A.fumigatus PDBID|CHAIN  LAPVAAALLG-----IGNLSGFDYRQLEQQRGSKISWYNAQFYNGWG----------------------------LAEDPRMYAAIVAQGWSPQ
2XVP:F|A.fumigatus CHIA1        SAAPQCIIPDAQLSDAIFNAAFDFIWIQYYNTAACSAKSFIDTSLGTFN---------------------------FDAWVTVLKASASKDA
3IAN:B|L.lactis Chitinase   su  MAPEFPYLTS--------SGKYAPYINNLDSYYD-FINPQYYNQGGDGFWDSDLN-----------------------MWISQSNDEKKEDFLYGLTQ
3N17:B|B.cereus Chitinase  NCT  MAPETAYVQGGYSAYGSIWGAYLPIIYGVKDKLT-YIHVQHFNAGSGIG--------------------------MDGNNYNQGTADYEVAMAD
3EBV:B|S.coelicolor Putative C  MAPQTIDMQ-------STQGGYFQTALNVKDILT-VVNMQYYNSGTMLG----------------------------CDGKVYAQGTVDFLTALAC

                                        510       520       530       540       550       560       570       580       590       600
                                ....|....|....|....|....|....|....|....|....|....|....|....|....|....|....|....|....|....|....|....|
3G6M:F|C.rosea Nematophagous f  KIILGMPIYGRAFVGTD--GPGKPYSTIG-----------------------------EGSWESGIWDYKVLPKAGATVITDSAAGATYSYDSSS----RT
1O6I:B|S.Marcescens CHITINASE   KIVMGVPFYGRAFKGVSGGNGGQYSSHSTPGEDPY----PSTDYWLVGCEECVRDKDPRIASYRQLEQMLQGNYGYQRLWNDKTKTPYLYHAQN----GL
3ARX:B|V.harveyi Chitinase A    KLVLGTAMYGRGWEGVTPDTLTDPNDPMTGTATG------KLKGSTAQGVWEDGVIDYKGIKSFMLGANNTGINGFEYGYDAQAEAPWVWNRST----GE
3OA5:B|Y.entomophaga Chi1 Chit  AIFIGYAGYT NA NATITTSIPSEEAL GTYTDAN--------QTLGSFEYSVLEWTDIICHYMDFE GEG NGY LVHDKVAKADYLYSEAT----KV
1ITX:B|B.circulans Chitinase A  KLVLGVPFYGRGWDGCAQAGNGQYQTCTG-----------------------GSSVGTWEAGSFDFYDLEANYINKNGYTRYWNDTAKVPYLYNASN----KR
1KFW:B|Arthrobacter sp. Psychr  QLGLGLAAYGRGWTGAKNVSPWGPATDGA-----------------------------PGTYETANEDYDKLKTLG-TDHYDAATGSAWRYDG------TQ
1WB0:M|H.sapiens HUMAN CHITINA  KLILGMPTYGRSFTLASSSDTRVGAPATGSGTPG-----------------------PFTKEGGMLAYYEVCSWKGATKQRIQDQKVPYIFRDN------Q
3QOK:B|K.pneumoniae Putative c  QMNLGIGFYGRVPKRAVEPGIDWTKA AQNNPVTQPYFGPQQIALFASLGY LSK TYVKYN IVGKLLN PQKRFT HW  AKVPWLSVQSA GKPLF
3ALF:P|N.tobaccum Class V Chit  KLVLGIPFYGYAWRLVNANIHGLRAPAAGKSNVG-----------------------AVDDGSMTYNRIRDYIVESRATTVYNATIVGDYCYSGS-----N
3SIM:P|C.vernus Family 18 Chit  KYKGG------------------------------------------------------NVLISFSTGPHPGGLPVDKGFFDAATSLKNKG------
3FND:B|B.thetaiotaomicron       KIVGGLPFYGYSWEESLQGAVDDVR-----------------------------------GIRYSGILKHLGNEAADKDNIGKTY-----------
2DSK:A|P.furiosus Hyperthermop  WKMIGLTP-------------------------------------------------------MIGVNDDKSVFTLEDAQQLVDWAIQHKIG-----
3BXW:M|H.sapiens Chitinase-Lik  PVLDGFSLMTYDYSTAHQPGPNAPLSWVRACVQVLD-----------------PKSKWRSKILLGLNFYGMDYATSKDAREPVVGARYIQTLKD----HR
2Y8V:F|A.fumigatus PDBID|CHAIN  RVVYG--------------------------------------------------------LLTNPGNGSQGYVPRERIGPVLAVLVEQFP------
2XVP:F|A.fumigatus CHIA1        KLYVGLP-------------------------------------------------------ASETAANQGYYLTPDEVESLVSTYMDRY------
3IAN:B|L.lactis Chitinase   su  RLVTG-------TDGFIKIPASKFVIGLP---------------------------------SNNDAAATGYVKDPNAVKNALNRLKAS-------
3N17:B|B.cereus Chitinase  NCT  MLLHGFPVGGNANNIFPALRSDQVMIGLP-------------------------------AAPAAAPSGGYISPTEMKKALNYIIKG--------
3EBV:B|S.coelicolor Putative C  IQLEG------------GLAPSQVGLGLP--------------------------------ASTRAAG-GGYVSPSVVNAALDCLTKA--------

                                        610       620       630       640       650       660       670       680       690       700
                                ....|....|....|....|....|....|....|....|....|....|....|....|....|....|....|....|....|....|....|....|
3G6M:F|C.rosea Nematophagous f  MISYDTPDMVRTKVSYAKGLGLGGSMFWEASADKTGSDSLIGTALSSMGSHDSTQNCLSYPNSKFDNIKNSLS---------------------------
1O6I:B|S.Marcescens CHITINASE   FVTYDDAESFKYKAKYIKQQQLGGVMFWHLGQDNRNGDLLAALDRYFNAADYDDSQLDMGTGLRYTGVGPGNLPIMTAPAYVPGTTYAQGALVSYQGYVW
3ARX:B|V.harveyi Chitinase A    LITFDDHRSVLAKGNYAKSLGLAGLFSWEIDAD--NGDILNAMHEGMAGGVVTPPNRRSHHHHHH-----------------------------------
3OA5:B|Y.entomophaga Chi1 Chit  FISLDTPRSVRDKGRYVKDKGLGGLFIWSGDQD--NGILTNAAHEGLKRRIKNKVIDMTPFYLDSDEELPTYTEPAEPQCEACNIK-------------
1ITX:B|B.circulans Chitinase A  FISYDDAESVGYKTAYIKSKGLGGAMFWELSGD-RNKTLQNKLKADL-----------------------------------------------------
1KFW:B|Arthrobacter sp. Psychr  WWSYDNIATTKQKTDYIVSKGLGGGMWWELSGDRNGE--LVGAMSDKFRAAAPGPVTEAAPP--------------------------------------
1WB0:M|H.sapiens HUMAN CHITINA  WVGFDDVESFKTKVSYLKQKGLGGAMVWALDLDDFAGFSCNQGRYPLIQTLRQELSLPYLPSGTPELEVPKPGQPSEPEHGPSPGQDTFCQGKADGLYPN
```

```
3QOK:B|K.pneumoniae Putative c  ALSY NPRSVAIKA YIKAKGLAGAMFWEYGADDQNQLARQLAESLGIKH--------------------------------------------------
3ALF:P|N.tobaccum Class V Chit  WISYDDTQTVRNKVNYVKGRGLLGYFAWHVAGDQNWGLSRTASQTWGVSFQEMK----------------------------------------------
3SIM:P|C.vernus Family 18 Chit  --------------------KLHGIAVWTADTSKSSDFRYEEEAQAFLVS--------------------------------------------------
3FND:B|B.thetaiotaomicron       ---YNGRPTIANKCKFIKENDYAGVMIWQLFQDAHNDNYDLKLINVVGREMMEEGHHHHHH---------------------------------------
2DSK:A|P.furiosus Hyperthermop  --------SLAFWSVDRDHPGPTGEVSPLHRGTNDPDWAFSHVFVKFMEAFGYTFSAQTSEASVPT----------------------------------
3BXW:M|H.sapiens Chitinase-Lik  PRMVWDSQASEHFFEYKKSRSGRHVVFYPTLKSLQVRLELARELGVGVSIWELGQGLDYFYDLL------------------------------------
2Y8V:F|A.fumigatus PDBID|CHAIN  --------------------NFGGVMGWEYFNSIPGEQQSPWQWAAEMSLSMHM----------------------------------------------
2XVP:F|A.fumigatus CHIA1        ------------------PDTFGGIMLWEATASENNQIDGAPYADHMKDILLH-----------------------------------------------
3IAN:B|L.lactis Chitinase   su  ------------------GNEIKGLMTWSVNWDAGTNSNGEKYNNTFVNTYAPMLFNNEGHHHHHH----------------------------------
3N17:B|B.cereus Chitinase  NCT  -VPFGGKYKLSNQSGYPAFR---GLMSWSINWDAKNNFEFSNNYRTYFDGLSLQK---------------------------------------------
3EBV:B|S.coelicolor Putative C  -TNCG-SFKPSKT--YPDLR---GAMTWSTNWDATAGNAWSNSVGAHVHALEGHHHHHH-----------------------------------------

                                        710       720       730
                                ....|....|....|....|....|....|....|.
3G6M:F|C.rosea Nematophagous f  ------------------------------------
1O6I:B|S.Marcescens CHITINASE   QTKWGYITSAPGSDSAWLKVGRVA------------
3ARX:B|V.harveyi Chitinase A    ------------------------------------
3OA5:B|Y.entomophaga Chi1 Chit  ------------------------------------
1ITX:B|B.circulans Chitinase A  ------------------------------------
1KFW:B|Arthrobacter sp. Psychr  ------------------------------------
1WB0:M|H.sapiens HUMAN CHITINA  PRERSSFYSCAAGRLFQQSCPTGLVFSNSCKCCTWN
3QOK:B|K.pneumoniae Putative c  ------------------------------------
3ALF:P|N.tobaccum Class V Chit  ------------------------------------
3SIM:P|C.vernus Family 18 Chit  ------------------------------------
3FND:B|B.thetaiotaomicron       ------------------------------------
2DSK:A|P.furiosus Hyperthermop  ------------------------------------
3BXW:M|H.sapiens Chitinase-Lik  ------------------------------------
2Y8V:F|A.fumigatus PDBID|CHAIN  ------------------------------------
2XVP:F|A.fumigatus CHIA1        ------------------------------------
3IAN:B|L.lactis Chitinase   su  ------------------------------------
3N17:B|B.cereus Chitinase  NCT  ------------------------------------
3EBV:B|S.coelicolor Putative C  ------------------------------------
```
