# Supplementary material for: Detection, Characterization and Evolution of Internal Repeats in Chitinases of Known 3-D Structure
Source: PLoS One. 2014 Mar 17;9(3):e91915. doi: 10.1371/journal.pone.0091915 (PMC3956812; doi:10.1371/journal.pone.0091915)
Supplement: Figure S2 — Multiple sequence alignment of 6 Endochitinase fold Chitinases with the repeats regions marked. (PDF) [file pone.0091915.s002.pdf]

Figure S2. Multiple sequence alignment of 6 Endochitinase fold Chitinases with the repeats regions marked with different color

```
                                  10        20        30        40        50        60        70        80        90       100
                          ....|....|....|....|....|....|....|....|....|....|....|....|....|....|....|....|....|....|....|....|
3CQL:P|C.papaya Family 19 Chit ----------------------------------------------------------GIEKIISRSMFDQMLKHRNNPACPAKGFYTYDAFIAAAKSFP
2Z37:P|B.juncea chitinase cata ----------------------------------------------------------DLSGIISRDQFYKMLKHMNDNDCHAVGFFTYDAFITAAKSFP
2DKV:P|O.sativa Class I Chitin --MEQCGAQAGGARCPNCLCCSRWGWCGTTSDFCGDGCQSQCSGCGPTPTPTPPSPSDGVGSIVPRDLFERLLLHRNDGACPARGFYTYEAFLAAAAAFP
3HBE:P|P.abies  Class IV Chiti ----------------------------------------------------------SVGGIISQSFFNGLAGG-AASSCEGKGFYTYNAFIAAANAYS
2CJL:B|S.coelicolor FAMILY 19 -------------------------------------------------------------FVVSEAQFDQMFPSRNS-------FYTYSGLTAALSAYP
1WVV:B|S.griseus Chitinase C m ATCATAWSSSSVYTNGGTVSYNGRNYTAKWWTQNERPGTSDVWADKGACGTGGEGPGGNNGFVVSEAQFNQMFPNRNA-------FYTYKGLTDALSAYP

                                 110       120       130       140       150       160       170       180       190       200
                          ....|....|....|....|....|....|....|....|....|....|....|....|....|....|....|....|....|....|....|
3CQL:P|C.papaya Family 19 Chit SFGTTGSTDVRKREIAAFLGQTSHETTGGWPSAPDGPYAWGYCFLKERNPS--SNYCAPS-PRYPCAPGK-SYYGRGPIQLSWNYNYGPCGEALRVNLLG
2Z37:P|B.juncea chitinase cata SFGNTGDLAMRKKEIAAFFGQTSHETTGGWSGAPDGANTWGYCYKEEIDKS--DPHCDSNNLEWPCAPGK-FYYGRGPMMLSWNYNYGPCGRDLGLELLK
2DKV:P|O.sativa Class I Chitin AFGGTGNTETRKREVAAFLGQTSHETTGGWPTAPDGPFSWGYCFKQEQNPP--SDYCQPS-PEWPCAPGR-KYYGRGPIQLSFNFNYGPAGRAIGVDLLS
3HBE:P|P.abies  Class IV Chiti GFGTTGSNDVKKRELAAFFANVMHETGG-------------LCYINEKNPP--INYCQSS-STWPCTSGK-SYHGRGPLQLSWNYNYGAAGKSIGFDGLN
2CJL:B|S.coelicolor FAMILY 19 GFSNTGSDTVKKQEAAAFLANVGHETGG-------------LVYVVEQNTANYPHYCDAS-QPYGCPAGNDKYYGRGPVQLSWNFNYKAAGDALGIDLLN
1WVV:B|S.griseus Chitinase C m AFAKTGSDEVKKREAAAFLANVSHQTGG-------------LFYIKEVNEANYPHYCDTT-QSYGCPAGQAAYYGRGPIQLSWNFNYKAAGDALGINLLA

                                 210       220       230       240       250       260       270       280       290       300
                          ....|....|....|....|....|....|....|....|....|....|....|....|....|....|....|....|....|....|....|
3CQL:P|C.papaya Family 19 Chit NPDLVATDRVISFKTALWFWMTPQAP-KPSCHDVITGRWQPSAADTAAGRLPGYGVITNIINGGLECGKGPNPQVADRIGFFRRYCGILGVGTGNNLDCY
2Z37:P|B.juncea chitinase cata NPDVASSDPVIAFKTAIWFWMTPQAP-KPSCHDVITDQWEPSAADISAGRLPGYGVITNIINGGLECAGRDVAKVQDRISFYTRYCGMFGVDPGSNIDCD
2DKV:P|O.sativa Class I Chitin NPDLVATDATVSFKTALWFWMTPQGN-KPSSHDVITGRWAPSPADAAAGRAPGYGVITNIVNGGLECGHGPDDRVANRIGFYQRYCGAFGIGTGGNLDCY
3HBE:P|P.abies  Class IV Chiti NPEKVGQDSTISFKTAVWFWMK-----NSNCHSAIT-----------SG--QGFGGTIKAIN-SMECNGGNSGEVSSRVNYYKKICSQLGVDPGANVSC-
2CJL:B|S.coelicolor FAMILY 19 NPDLVQNDSAVAWKTGLWYWNTQTGPGTMTPHDAMVNG-------------AGFGETIRSINGSLECDGGNPGQVQSRIDNYERFTQLLGVEPGGNLSC-
1WVV:B|S.griseus Chitinase C m NPYLVEQDPAVAWKTGLWYWNSQNGPGTMTPHNAIVNN-------------AGFGETIRSINGALECNGGNPAQVQSRINKFTQFTQILGTTTGPNLSC-

                                 310
                          ....|....|....|.
3CQL:P|C.papaya Family 19 Chit NQRPFG----------
2Z37:P|B.juncea chitinase cata NQRPFN----------
2DKV:P|O.sativa Class I Chitin NQRPFNSGSSVGLAEQ
3HBE:P|P.abies  Class IV Chiti ----------------
2CJL:B|S.coelicolor FAMILY 19 ----------------
1WVV:B|S.griseus Chitinase C m ----------------
```
