# Supplementary figures and images for: Detection, Characterization and Evolution of Internal Repeats in Chitinases of Known 3-D Structure
Source: PLoS One. 2014 Mar 17;9(3):e91915. doi: 10.1371/journal.pone.0091915 (PMC3956812; doi:10.1371/journal.pone.0091915)

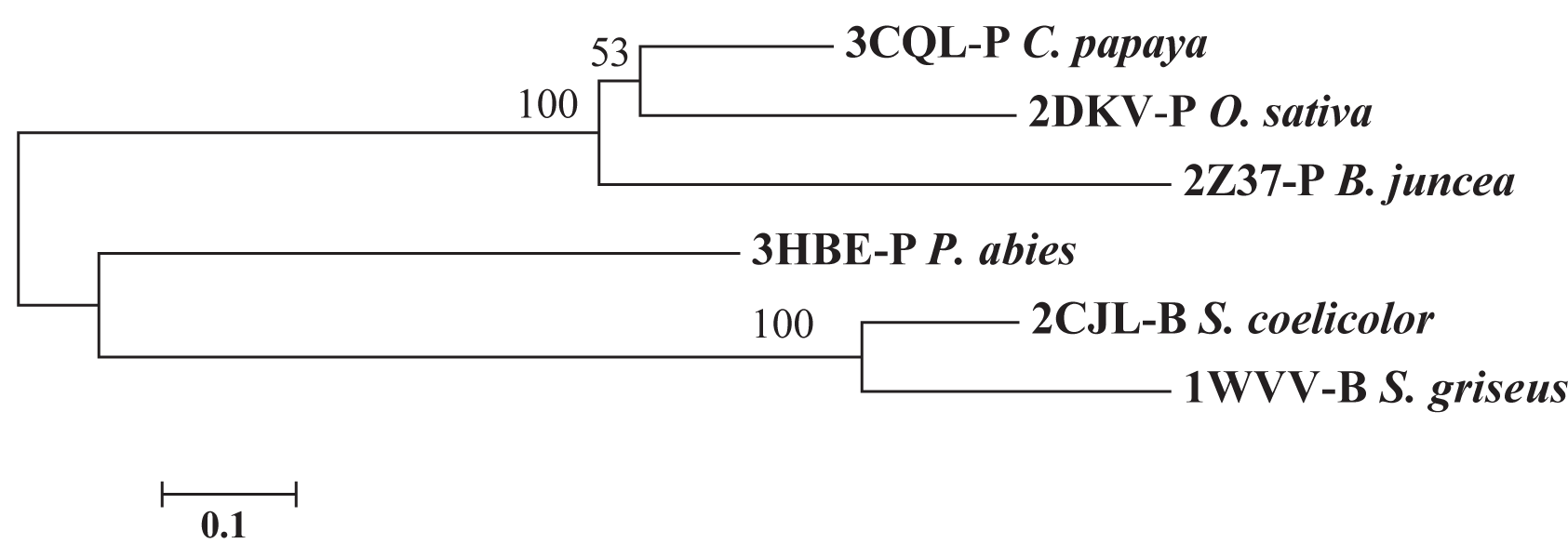

Supplement: Figure S3 — Phylogenetic relationship of Endochitinase fold Chitinases. Bootstrap support value (%) >50 is showed above branch. (TIF) [file pone.0091915.s003.tif]

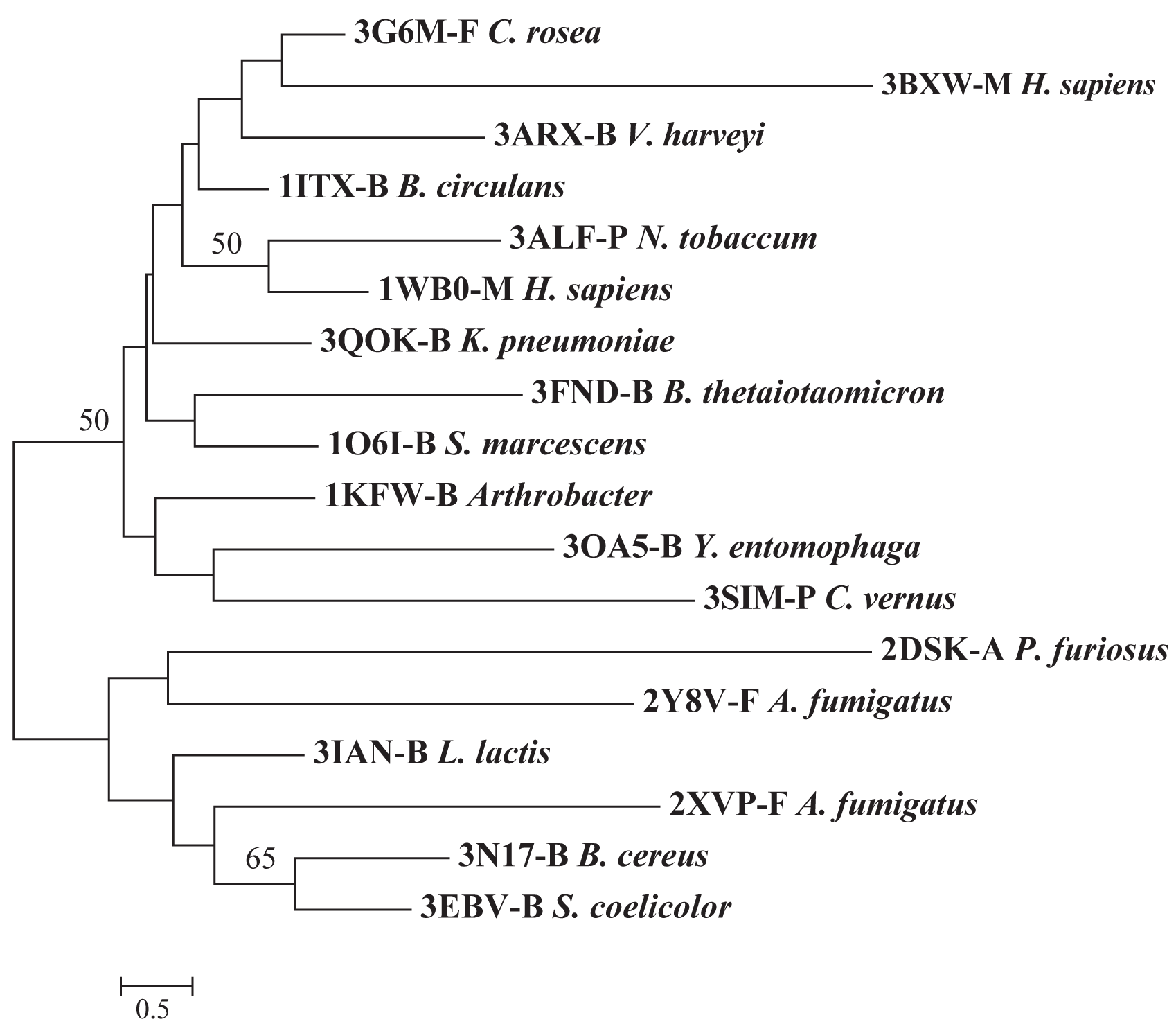

Supplement: Figure S4 — Phylogenetic relationship of TIM barrel fold Chitinases. Bootstrap support value (%) >50 is showed above branch. (TIF) [file pone.0091915.s004.tif]
