# Supplementary material for: Detection, Characterization and Evolution of Internal Repeats in Chitinases of Known 3-D Structure
Source: PLoS One. 2014 Mar 17;9(3):e91915. doi: 10.1371/journal.pone.0091915 (PMC3956812; doi:10.1371/journal.pone.0091915)
Supplement: Table S1 — Alignment scores of different pairs of Chitinases. (PDF) [file pone.0091915.s005.pdf]

Table S1. RMSD and Z-scores of structural superposition of proteins belonging to the TIM fold

	1O6I		3ARX		3OA5		1ITX		1KFW		1WB0		3QOK		3ALF		3SIM		3FND		2DSK		3BXW		2Y8V		2XVP		3IAN		3N17		3EBV	
	R	Z	R	Z	R	Z	R	Z	R	Z	R	Z	R	Z	R	Z	R	Z	R	Z	R	Z	R	Z	R	Z	R	Z	R	Z	R	Z	R	Z
3G6M	1.48	17.5	1.44	19.8	1.58	16.3	1.20	20.6	1.32	17.8	1.50	17.9	1.67	15.8	1.80	17.6	2.44	8.6	2.00	12.9	2.57	7.8	2.24	10.3	2.67	11.8	2.33	11.5	2.49	10.1	2.49	10.8	2.21	12.0
1O6I			1.76	16.8	1.85	14.0	1.46	17.8	1.70	15.9	1.58	17.1	1.78	15.4	1.74	16.4	2.71	7.8	1.97	12.4	2.48	7.6	2.05	10.0	2.85	10.0	2.58	9.8	2.90	6.6	2.62	10.2	2.31	10.9
3ARX					1.63	16.9	1.37	20.6	1.54	17.0	1.60	17.8	1.86	16.1	1.88	16.4	2.71	7.4	1.88	13.7	2.70	6.5	2.31	10.7	2.56	10.2	2.50	10.4	2.63	9.5	2.65	10.4	2.32	10.7
3OA5							1.54	17.8	1.69	15.9	1.76	15.3	1.96	15.4	1.93	15.2	2.83	7.3	2.18	12.2	2.67	7.3	2.41	9.7	2.70	11.1	2.71	9.5	3.06	8.1	2.86	9.7	2.38	10.7
1ITX									1.32	17.6	1.34	19.7	1.56	18.3	1.57	17.6	2.55	8.0	1.83	14.0	2.60	7.1	2.17	11.2	2.81	9.7	2.35	10.6	2.61	9.2	2.64	10.6	2.29	10.8
1KFW											1.77	15.7	1.69	15.0	1.78	15.1	2.61	7.3	2.26	12.5	2.66	6.6	2.34	8.4	2.45	10.0	2.63	10.2	2.81	9.0	2.62	9.6	2.45	9.7
1WB0													1.49	17.0	1.37	19.3	2.51	7.7	1.87	13.6	2.45	6.7	2.04	11.1	2.60	10.8	2.48	10.3	2.54	8.9	2.49	9.9	2.36	10.6
3QOK															1.57	15.8	2.35	8.2	1.75	14.0	2.43	7.7	2.32	10.5	2.51	11.6	2.49	10.3	2.60	8.7	2.33	10.3	2.19	11.0
3ALF																	2.58	7.1	1.71	12.9	2.43	8.0	2.15	10.6	2.87	10.7	2.53	10.0	2.59	8.3	2.57	10.1	2.36	10.6
3SIM																			2.63	7.2	2.65	5.6	2.86	5.8	2.49	7.8	2.39	8.4	2.30	9.5	2.14	10.0	2.34	9.5
3FND																					2.19	7.2	2.16	8.2	3.06	8.5	2.65	8.3	2.48	7.5	2.27	9.7	2.41	8.9

**2DSK**																							**2.49**	**6.0**	**2.66**	**6.7**	**2.94**	**6.0**	**2.46**	**5.8**	**2.49**	**6.4**	**2.46**	**7.0**
**3BXW**																									**2.63**	**8.0**	**2.78**	**6.9**	**3.08**	**6.6**	**2.71**	**7.9**	**2.40**	**8.4**
**2Y8V**																											**2.62**	**11.6**	**2.37**	**10.0**	**2.22**	**12.0**	**2.16**	**12.0**
**2XVP**																													**2.22**	**10.8**	**2.01**	**12.6**	**2.02**	**12.8**
**3IAN**																															**1.63**	**14.8**	**1.80**	**13.7**
**3N17**																																	**1.44**	**16.6**

R- RMSD values; Z- Z Scores
