# Supplementary material for: Detection, Characterization and Evolution of Internal Repeats in Chitinases of Known 3-D Structure
Source: PLoS One. 2014 Mar 17;9(3):e91915. doi: 10.1371/journal.pone.0091915 (PMC3956812; doi:10.1371/journal.pone.0091915)
Supplement: Table S3 — RMSD and Z-scores of structural superposition of proteins belonging to the Endochitinase fold. (PDF) [file pone.0091915.s007.pdf]

Table S3. Alignment scores of different pairs of Chitinase

	3FND	3G6M	3HBE	3IAN	3N17	3QOK	3ALF	3ARX	3BXW	2Z37	2Y8V	2XVP	2D49	2DKV	2DSK	2CWR	2CJL	1VF8	1WVV	1ED7	1ITX	1K85	1KFW	1O6I	1CNV	1GOI	1HP5	1WB0	2DQA	2HVM	2UY2	3CQL	3EBV	3OA5
3G6M	12.5																																	
3HBE	12.7	13.7																																
3IAN	15.3	10.5	13.7																															
3N17	12.1	10.8	14.2	23.3																														
3QOK	13.7	19.9	12.2	11.8	14.7																													
3ALF	12.5	22.3	12.7	14.0	12.3	17.2																												
3ARX	12.5	17.9	11.7	12.4	11.4	17.3	14.7																											
3BXW	11.2	11.9	10.7	9.6	10.8	9.9	10.1	11.7																										
2Z37	10.6	10.6	36.2	10.2	10.6	11.0	11.4	11.8	10.2																									
2Y8V	10.3	11.0	12.7	12.0	11.7	12.7	12.4	11.3	10.0	9.4																								
2XVP	11.2	13.5	13.2	10.9	12.2	11.6	10.6	12.5	10.6	10.6	11.7																							
2D49	15.0	22.6	18.8	15.1	16.9	16.9	22.6	16.9	15.0	18.8	16.9	16.9																						
2DKV	8.7	11.9	29.2	11.0	11.0	11.0	11.3	11.6	9.7	57.7	11.3	11.0	18.8																					
2DSK	11.2	11.8	12.2	12.5	10.9	13.5	102	11.8	11.2	9.0	12.0	9.6	16.9	9.7																				
2CWR	12.6	13.5	11.6	14.5	13.5	14.5	13.5	16.5	14.5	13.5	18.4	14.5	15.0	16.5	13.5																			
2CJL	11.7	12.2	41.1	12.2	13.2	13.2	13.7	14.2	10.7	32.3	11.7	12.2	15.0	37.2	11.7	14.5																		
1VF8	13.7	17.7	11.7	12.7	10.2	18.5	23.7	13.5	10.0	10.6	14.1	10.6	15.0	10.6	11.2	15.5	11.7																	
1WVV	11.3	13.2	39.2	12.1	11.6	12.8	12.0	12.8	10.9	22.1	9.8	10.1	98.1	29.4	10.5	15.5	76.4	10.1																
1ED7	13.3	24.4	17.7	15.5	17.7	20.0	17.7	20.0	20.0	13.3	15.5	15.5	24.1	15.5	20.0	15.5	17.7	17.7	24.4															
1ITX	12.1	27.5	11.7	11.8	12.9	22.6	22.1	28.1	10.9	11.4	13.4	10.9	18.8	11.6	11.2	14.5	12.2	22.0	15.0	17.7														
1K85	14.7	19.3	15.9	17.0	14.7	15.9	18.1	19.3	17.0	14.7	12.5	13.6	22.6	18.1	15.9	11.3	14.7	13.6	15.9	17.7	21.5													
1KFW	10.8	21.6	12.2	12.7	12.3	18.3	17.2	15.4	11.0	13.1	11.0	14.1	20.7	12.6	10.9	17.4	13.2	16.1	14.7	17.7	26.4	15.9												
1O6I	13.1	19.7	12.2	11.2	12.0	18.1	16.7	11.0	10.9	11.8	13.1	10.9	26.4	10.6	11.5	13.5	11.2	17.5	10.9	22.2	24.8	15.9	13.3											
1CNV	9.6	13.7	11.7	11.7	14.7	12.7	11.3	12.0	11.0	9.8	12.7	20.4	18.8	12.0	12.0	11.6	10.2	11.3	9.8	15.5	11.7	15.9	12.0	11.0										
1GOI	13.1	19.4	12.2	11.2	11.7	17.8	16.4	11.0	10.9	11.8	12.4	10.9	26.4	10.6	11.5	13.5	12.2	17.7	10.9	22.2	24.5	15.9	13.3	99.7	11.0									
1HP5	10.5	11.5	11.7	10.9	13.5	11.1	12.1	10.7	10.6	11.8	12.7	11.6	16.9	11.9	11.8	15.5	11.2	10.6	11.3	20.0	12.4	17.0	12.4	12.0	11.3	12.0								
1WB0	13.4	19.7	12.7	11.2	14.7	17.3	26.6	13.0	12.4	12.2	14.3	10.6	16.9	11.6	12.5	12.6	12.7	46.1	12.4	17.7	24.8	18.1	14.2	19.3	12.3	19.1	11.6							
2DQA	12.9	12.1	14.5	12.1	12.1	12.1	11.2	12.0	12.9	12.1	13.7	14.5	15.0	13.7	12.0	10.6	13.7	12.9	14.5	13.3	12.0	9.0	12.9	12.9	12.0	12.9	14.5	12.0						
2HVM	12.4	13.1	12.7	16.4	16.8	11.7	12.8	12.4	11.3	11.0	15.3	23.8	16.9	11.7	10.6	12.6	10.2	10.9	13.2	17.7	13.1	17.0	13.1	15.7	32.9	15.3	10.9	13.5	13.7					
2UY2	10.8	14.6	13.2	16.6	15.3	12.2	10.5	12.9	10.8	10.6	12.7	28.5	16.9	12.5	11.9	12.6	14.7	11.2	12.0	24.4	12.2	15.9	13.6	12.2	20.0	11.9	12.2	11.9	12.0	29.6				
3CQL	11.9	12.7	42.1	11.5	12.3	12.7	11.1	13.5	11.5	64.6	12.7	9.8	22.6	72.0	9.8	15.5	36.2	11.5	31.6	17.7	11.5	19.3	12.3	11.5	9.8	11.5	12.3	11.1	12.0	10.6	13.1			
3EBV	12.5	11.9	14.2	18.2	28.1	14.9	13.5	13.5	12.9	11.4	13.7	13.9	16.9	10.2	12.9	15.5	13.2	10.9	12.0	20.0	13.5	18.1	13.9	14.9	12.7	14.5	11.5	13.5	12.9	15.7	18.0	11.9		
3OA5	11.2	19.9	11.2	12.7	12.9	15.7	16.7	16.0	12.2	11.8	11.7	10.9	15.0	11.3	11.2	14.5	12.7	14.3	11.3	15.5	20.5	20.4	14.4	12.2	12.3	12.2	11.5	13.0	12.0	11.3	12.2	14.4	11.9	
3SIM	13.4	11.2	11.2	12.7	13.1	15.2	12.0	12.0	11.6	11.4	12.7	13.4	16.9	9.0	12.7	13.5	11.7	11.2	10.5	22.2	13.0	18.1	12.7	13.4	11.6	13.4	11.6	12.0	12.9	13.5	13.8	10.6	14.5	11.2
