# Supplementary material for: Detection, Characterization and Evolution of Internal Repeats in Chitinases of Known 3-D Structure
Source: PLoS One. 2014 Mar 17;9(3):e91915. doi: 10.1371/journal.pone.0091915 (PMC3956812; doi:10.1371/journal.pone.0091915)
Supplement: Table S2 — RMSD and Z-scores of structural superposition of proteins belonging to the TIM fold. (PDF) [file pone.0091915.s006.pdf]

Table S2. RMSD and Z-scores of structural superposition of proteins belonging to the Endochitinase fold

	2Z37		2DKV		3HBE		2CJL		1WVV	
	RMSD	Z Score	RMSD	Z Score	RMSD	Z Score	RMSD	Z Score	RMSD	Z Score
3CQL	0.87	15.1	1.02	15.1	1.40	11.4	1.7	10.4	1.51	11.5
2Z37			0.70	16.3	1.29	10.7	1.23	12.1	1.05	13.0
2DKV					1.05	13.0	1.25	11.8	1.32	12.3
3HBE							3.34	2.7	3.11	2.7
2CJL									0.83	13.6
